# Component-Resolved Diagnosis in Pediatrics

**DOI:** 10.5402/2012/806920

**Published:** 2012-08-05

**Authors:** Ole D. Wolthers

**Affiliations:** Asthma and Allergy Clinic, Children's Clinic Randers, Dytmærsken 9, 8900 Randers, Denmark

## Abstract

Component resolved diagnosis is a new concept in the investigation of pediatric allergic disease. The aim of the present paper is to review the available data on component resolved diagnosis with respect to implications for investigation of children with allergic disease. In most conditions head-to-head comparisons of component resolved diagnosis with traditional IgE testing have not been performed. Rather than alternatives the molecular methods should be seen as adjuncts to the cheaper traditional specific IgE tests. It may be appropriate to determine IgE antibodies to components as part of the diagnostic work-up in selected cases of peanut and birch pollen allergy and in hymenoptera allergy. However, cost benefit analyses of component resolved diagnosis compared with traditional work-up of allergy are needed. Prospectively planned protocols for assessment of the extent to which component resolved diagnosis may be able to improve the selection of children to immunotherapy and, thus, the efficacy of immunotherapy, are needed. Finally, studies of component resolved diagnosis with microarray technology in screening panels with hundreds of components should be undertaken before it can be determined to which extent such panel screening, if at all, may be helpful in children.

## 1. Introduction

Traditionally, diagnosis of pediatric allergic disease is based on a careful and thorough history, skin prick testing, and assessment of specific IgE antibodies to allergens in the blood, and on provocation or elimination-provocation-elimination tests. By means of molecular methods over the last few years, it has become possible to measure IgE antibodies to specific components of allergens. The methods are designated as component-resolved diagnosis [1–4].The molecular structures of many allergens have been characterized and are commercially available as recombinant products. This has focused attention on the need for assessment of the clinical application of the methods in pediatric populations; however, guidelines or consensus on their use have not been defined. The aims of this paper is to introduce the concept of component resolved diagnosis, to identify conditions in which the new diagnostic tool may be helpful in pediatric allergic disease, and to discuss conditions in which more evidence should be provided before large-scale use of the methods may be warranted.

## 2. Allergens and Components

All protein component material possesses a potential for development of allergy. Proteins in *allergen sources* (e.g., peanuts, pollen, and hymenoptera) often contain several different allergenic parts, *allergen components*, which contain different *epitopes*, that is, peptides, which are three-dimensional binding sites for corresponding IgE antibodies. There is no general feature of the epitopes which determine whether they have allergic effects or not. Some epitopes are species-specific for their allergen source, for instance, cats; others are so similar to each other that they may constitute “*epitope families.*” Some epitopes in birch pollen resemble the epitopes in peanuts, hazelnuts, and other stone and leguminous fruits. That causes what is often referred to as *cross-reactivity.*

 Based on two other important epitope features, they are divided into *heat stable* (as opposed to heat labile) and *digestion stable* (as opposed to digestion labile epitopes they resist ventricle denaturation). The more stable to heat and digestion an epitope, the higher the risk of serious clinical symptoms in the sensitized patient when exposed to the allergen. Differences in epitope family relations and heat and digestion stability often explain differences in allergenicity and clinical presentations. For example, a genuine peanut IgE-mediated allergy often causes more serious symptoms than if an IgE positive peanut test is due to a cross-reactivity to birch pollen because the epitopes in the “genuine” allergy are not denatured to the same extent as the epitopes involved in the cross-reactivity. One of the strengths of component resolved diagnosis is that in some situations it may be able to discriminate between genuine allergy and cross-reactivity in patients, in whom the differentiation is difficult to establish from symptoms and signs. 

*Allergen sources *are named after their Latin family names. For example, the Latin designation of peanut is *Arachis hypogaea*. This allergen source contains a range of different *allergen components *each of which is identified by numbers: 1, 2, 3, and so forth. Traditionally, abbreviations of the Latin names are used. Thus, *Ara h1* designates allergen number 1 from *Arachis hypogaea*, that is, peanut. 

 In the following, the analyses which are relevant to consider at present are marked with an ∗. 

## 3. Food Allergy

### 3.1. Peanut (*Arachis Hypogaea*)

Components: Ara h1–storage protein (7S globulin); Ara h2-storage protein (2S albumin)*; Ara h3-storage protein (11S globulin); Ara h8-Bet v 1 (birch) homologue∗; Ara h9-lipid transfer protein (LTP).

Component resolved diagnosis is valuable in the investigation of peanut allergy in children in whom it may be difficult to decide whether a sensitization may be caused by genuine allergy or a cross-reactivity. In such cases there is good evidence for analyzing IgE to Ara h2 (genuine IgE-mediated allergy) and Ara h8 (Bet v 1 (birch pollen) homologue; a marker of cross-reactivity) [5–7]. IgE sensibilization to Ara h2 often correlates with positive IgE against Ara h1 and Ara h3. If there are IgE antibodies in serum to Ara h2 and/or Ara h1/Ara h3, more than 95% of the patients will have symptoms when ingesting peanuts [8]. If there is IgE only to Ara h2 and not to Ara h1, 3 or 8 87% report symptoms. Whether there may be a threshold level of IgE to Ara h 2 above which peanut allergy may be diagnosed with a sufficient clinically sensitivity and specificity which may abandon the need for oral provocation remains to be evaluated. If there is only IgE to Ara h8 and not to Ara h1, 2 or 3 only around 18% of children report allergy symptoms, and these are usually very mild [9]. More serious symptoms cannot be ruled out, however, in Ara h8 sensitized patients. It is still unclear how stable the component actually is against ventricular enzymes, and how much of the allergen may pass unaffected through the stomach. In the event of itching and swelling in the mouth and throat sensitization to Ara h2 cannot, however, be completely ruled out. In such cases, both Ara h2 and Ara h8 should be determined, and, at the same time, assessment of sensitization to birch pollen should be made by analyzing IgE antibodies to Bet v 1 [2]. 

### 3.2. Hazelnut (*Corylus avellana*)

Components: Cor a1-Bet v 1 (birch) homologue*; Cor a8-lipid transfer protein (LTP)*.

Increased IgE to hazelnut often reflects cross-reactivity. When investigating allergy to hazelnut, sensitization to birch should be looked for in order to relate to a possible sensitization to Cor a1 and Cor a8 [10, 11]. A high Cor a1 is usually explained by an even higher IgE level to birch pollen. Sometimes, the sum of Cor a 1 and Cor a8 is lower than for the hazelnut allergen. In such cases, patients probably have IgE antibodies to other protein components than Cor a1 and Cor a8 in hazelnuts, which cannot yet be analyzed. 

### 3.3. Wheat (*Triticum aestivum*)

Component: Omega-5 gliadin.

The prevalence of allergy to wheat varies throughout the world as does the prevalence of asymptomatic sensitization. There is a significant cross-reactivity between wheat and grass pollen which leads to overdiagnosing of wheat allergy [12]. IgE antibodies to omega-5 gliadin are associated with genuine wheat allergy and when in doubt about whether a genuine allergy is present or not one may consider to assess omega-5 gliadin [13]. If omega-5 gliadin is increased, it is more probable that the condition is due to wheat allergy than to cross-reactivity [14]. As IgE antibodies against omega-5 gliadin may provide more reliable information than determination of IgE antibodies to the allergen, one may also consider testing for omega-5 gliadin if there is both a reaction to wheat-containing food ingested few hours before a clinical reaction and normal IgE levels to the allergen [1]. If increased omega-5 gliadin values are found in that situation, one may consider to perform a wheat challenge [2]. More evidence, however, is needed before firm recommendations can be given. 

### 3.4. Soy (*Glycine Max*)

Components: Gly m4-Bet v1 (birch) homologue; Gly m5; Gly m6.

Soy allergy is mostly seen in children who have received soya-based milk substitute products. The most important markers of soy allergy are Gly m5 and 6 [15]. There is, however, a significant cross-reactivity associated to soy which explains most elevated specific IgE values to the allergen. Of the patients who have IgE antibodies to peanuts (Ara h 2), about 60% also have IgE antibodies to soy protein [16]. In these cases, the sensitization to soy should be regarded as a cross-reactivity since the vast majority of patients (95%) present no symptoms when having ingested soy. Gly m 4, a birch pollen homologue (Bet v 1), will be positive indicating the cross-reactivity. Therefore, if an allergic reaction to soy is suspected and specific IgE to soy < 0,35 kU/L, IgE antibodies to Gly m 4 and IgE to birch pollen should be assessed. Very little Gly m 4 occurs in the test when specific IgE to soy is determined, so, IgE to the allergen may be negative even if IgE antibodies to Gly m 4 are elevated. 

#### 3.4.1. Wheat, Soy, and Exercise Induced or Idiopathic Anaphylaxis 

Rare, severe anaphylactic reactions to wheat and soy, which can be attributed to omega-5 gliadin (wheat component) and Gly m 4 (soy component), may be seen in runners after ingestion of wheat-containing food and in birch pollen-sensitized runners during the birch pollen season who, after physical exercise, have drunk highly concentrated soy drinks [17, 18]. In anaphylaxis with or without a suspected relation to exercise in which trigger mechanisms are unknown, one should therefore consider determining IgE antibodies to omega-5 gliadin and Gly m 4, if specific IgE for the wheat and soy allergens is <0,35 kU/L and wheat and soy cannot be excluded as etiological factors. At the same time, specific IgE antibodies against birch pollen should be analyzed.

### 3.5. Egg (*Gallus Domesticus*)

Components: Gal d1-ovomucoid; Gal d2-ovalbumin; Gal d3-conalbumin;Gal d4-lysozyme.

Egg white is the most important allergen source in hens' eggs. Gal d1 comprises only approximately 10% of the protein content in egg white, but appears to be the most important allergen component [19]. High concentrations of Gal d1 are associated with persistent allergy to eggs. It has been suggested that the lower the Gal d 1 concentration, the higher the probability of tolerance to cooked egg [20, 21]. As Gal d 1 appears to be associated with persistent allergy to eggs, one may consider assessing Gal d 1 for prognostic reasons. It has also been suggested that IgE antibodies to the Gal d 1 component may be assessed when IgE levels against the allergen are low with the aim of guidance on whether or not oral provocation should be performed [20]. More evidence is needed before firm conclusions can be drawn. 

### 3.6. Milk

Components: Bos d4, Alpha-lactoglobulin; Bos d5, Beta-lactoglobulin; Bos d8, Casein; Bos d lactoferrin.

Milk-allergic children are often sensitized to several cow milk proteins, and no single component seems to be playing a central role [1, 2]. Preliminary studies have suggested that children who outgrow their milk allergy have heat labile epitopes, whereas the relatively few children in whom the milk allergy persists appear to have heat stable epitopes. High IgE antibody levels to casein may be associated with persistent milk allergy [22]. There is, however, no clarification of this yet.

## 4. Pollen Allergy

### 4.1. Grass Pollen (*Phleum pratense* (Timothy Grass))

Components: Phl p1; Phl p5; Mix Phl p1/Phl p5; Phl p4; Phl p7; Phl p12 Profilin.

IgE antibodies to Phl p 1 and Phl p 5 are specific markers for sensitization to Timothy grass. Phl p 7 (calcium-binding protein) and Phl p 12 (profilin) are markers of cross-reactivity. Increased IgE to these components and not to Phl p 1 and/or Phl p 5 indicates primary sensitization to another pollen [23].

 Specific immunotherapy is the only treatment modality which implies an immunomodulating potential. For unknown reasons, immunotherapy is not effective in all *Phleum pratense* sensitized, symptomatic patients. Therefore, attention has been focused on whether component resolved diagnosis may improve the selection of patients to immunotherapy. It has been suggested that if relevant symptoms are present in addition to elevated IgE Phl p 1 and p 5 levels immunotherapy with *Phleum pratense* extract would probably be clinically effective [1, 24]. Whether the strategy may increase the effect ratio of immunotherapy has not, however, been tested in prospectively planned trials.

### 4.2. Birch Pollen (*Betula verrucosa*)

Components: Bet v1, PR-10 protein*; Bet v2, profilin*; Bet v4, calcium-binding protein. 

Bet v1, PR-10 protein is the major allergen in birch pollen and around 95% of birch pollen-sensitized patients have specific IgE antibodies to Bet v1 [25, 26]. It has been suggested that immunotherapy with birch pollen extract will probably be clinically effective if there are characteristic symptoms during the birch pollen season in Bet v 1 IgE-positive patients [1]. Whether the selection of patients for immunotherapy based on Bet v 1 may increase the success rate has not been tested, however. The question is complicated by the fact that specific IgE to Bet v 1 may also be found in patients with a primary sensitization to other tree pollens (e.g., elm: Aln g1; hazel pollen: Cor a1) and to foods (hazelnut, apple, soy, peanut (Ara h8), kiwi, and celery) [25–28]. 

 IgE antibodies to Bet v 2, profilin and/or Bet v 4, calcium-binding protein are markers of cross-reactivity [25]. If increased IgE to these components, but not to Bet v 1, are detected the patient is probably primarily sensitized to another pollen. IgE to Bet v 2 is a marker of cross-reactivity between many pollens and vegetables [29]. IgE to Bet v 4 is a marker of cross-reactivity only between pollen allergens [30]. 

### 4.3. Mugwort (*Artemisia vulgaris*)

Components: Art v1; Art v3.

IgE antibodies to Art v 1 and Art v 3 have been identified as specific allergen markers. This has been taken as an indication that immunotherapy with mugwort extract will probably be clinically effective in symptomatic patients [1]. Again, whether a decision to initiate immunotherapy based on component-resolved diagnosis versus specific IgE to the allergen will improve the efficacy of immunotherapy has not been rigorously assessed. 

## 5. House Dust Mite Allergy

### 5.1. House Dust Mites (*Dermatophagoides pteronyssinus; Dermatophagoides farinae*)

Components: Der p1, cysteine protease; Der p2, NPC2 family; Der p10, tropomyosin.

Der p 1 and Der p 2 are the most important component markers for sensitization to house dust mites [31]. More than 80–90% of patients allergic to house dust mites have IgE antibodies to the epitopes. Approximately 10% of patients allergic to house dust mites, however, have increased IgE levels to Der p 10, tropomyosin which appears to cross-react with prawn and cockroach [32]. Whether component resolved diagnosis may improve management of children with house dust mite allergy remains to be elucidated. 

## 6. Animal Hair Allergy

### 6.1. Cat (*Felis domesticus*)

Components: Fel d1; Fel d2, serum albumin.

Around 60–90% of patients with allergy to cat have IgE antibodies against Fel d 1 [33]. It has been suggested that this finding may be interpreted as an indicator of effect of immunotherapy as the extract primarily contains Fel d 1 [1]. Whether that holds water remains to be seen. 

 Fel d 2, serum albumin cross-reacts with a range of other furred animals. It can also react with pork (and cause the rare so-called “cat-pork syndrome”) [34]. Approximately 15–40% of cat-allergic patients have IgE antibodies to Fel d 2.

### 6.2. Dog (*Canis familiaris*)

Components: Can f1; Can f2; Can f3, serum albumin; Can f4, Can f5, male dog urine protein.

Can f1, Can f2, Can f4, and Can f5 are specific components which indicate primary sensitization [35]. Around 50–90%, 20–33% and 70% of patients allergic to dogs have IgE antibodies to Can f 1, 2, and 5, respectively [35, 36]. Whether the components may be more prevalent in some dog breeds than others, which would explain why some patients with dog hair allergy tolerate contact with some dog breeds better than with others, has not yet been explored. The presence of IgE antibodies against Can f 1 has been suggested to be a predictor of immunotherapy efficacy, however, that has not been clarified yet [1]. Can f 3 is a cross-reacting component which reacts with other furred animals such as cat and horse [37]. 

### 6.3. Horse (*Equus caballus*)

Components: Equ c 1; Equ c 3.

Equ c 1 is assumed to be the primary component in horse dander [38]. The cat component Fel d 4 has cross-reactivity with Ecu c 1 and may give rise to increased concentration of IgE to the allergen which may be incorrectly interpreted as an indication of horse hair allergy. Whether component resolved diagnosis may improve management of children with allergy to horse also remains to be evaluated. 

## 7. Hymenoptera Allergy

### 7.1. Bee (*Apis mellifera*) and Wasp (*Vespula vulgaris*)

Components: Api m 1, bee∗; Ves v 5, wasp∗; Ves v 1, wasp; Pol d 5, paper wasp.

Several major glycoprotein components have been identified in the venom of bee and wasp. The most important specific major components are Api m 1 (phospholipase A2) in the bee, Ves v 1 (phospholipase A1) and Ves v 5 (antigen 5) in the wasp [39]. The major components are quite similar and to a great extent the structure of phospholipase also is similar in different bees. Antigen 5 is the major allergen in all wasp venom.

 Positive IgE to both bee and wasp venom is often due to cross-reactivity to cross-reactive carbohydrate-determining reagents (CCD) [40]. In the frequently occurring clinical situation of an uncertain history and positive IgE to both the allergens, Api m 1 and Ves v 5 IgE should be determined. An increase in both Api m 1 and Ves v 5 would indicate a true double sensitization, and immunotherapy with both bee and wasp extracts would be indicated [39, 40]. 

## 8. Discussion

By no means does this paper aim at reviewing all components that have been detected so far. More than 100 epitopes have been identified [2]. The clinical relevance of many of them is not known. No doubt, in the future many more will see the light of day. The development of *microarray technology, *which enables easy and speedy screening of hundreds of components in few *μ*L of blood (as little as 20 *μ*L; conventional specific IgE tests use 50 *μ*L per allergen) has a potential of an extremely detailed and differentiated diagnosis [23]. From the industry, of course, there is a commercially motivated pressure for making use of the new screening opportunities, and effort is being put into developing electronic programs which will help clinicians to interpret screening panels containing hundreds of allergen components [2]. No doubt, the microarray technology creates new possibilities for research in the development of IgE-mediated allergy, cross-reactivity, prognosis, effects of specific and patient-individualized allergen avoidance procedures, dietetic, prophylactic, and therapeutic treatments. At the same time, however, there is a significant risk that we will be faced with a data complexity which may challenge clinicians' specific knowledge in the area. Misinterpretations and overdiagnosis may knock on our door [1, 2, 41]. No less now than before it is necessary to remember that the clinical significance of each discovered sensitization can only be assessed in relation to history and signs. Sensitization is not synonymous with disease. Screening with IgE test panels with higher numbers of allergens have been found far from always to improve diagnostic sensitivity and specificity [42]. In many cases we may be better off by only performing the allergen component test which is indicated by the history and the specific clinical situation [42]. In the investigation of food allergy it may be possible that screening tests consisting of large panels of components will be helpful because it can often be difficult to carry out food challenges, especially, if there is a suspicion of many relevant significant allergen sources. The question, however, needs rigorous evidence testing before screening panels should be recommended for use in clinical practice. Similarly, cost-benefit analyses of component resolved diagnosis compared with traditional workup of food allergy including oral provocation tests are still needed. The molecular methods cannot replace oral provocation tests. 

At present there are no clinical guidelines or consensus about the application of component resolved diagnosis in children or, indeed, in adults. To some extent that may be due to insufficient data on the use of the methods as documented above. Furthermore, it has not been decided which level of specialized qualifications clinicians should have to be allowed to order the analyses and to interpret and initiate treatments on the basis of the results. It would seem appropriate to restrict their use to fully trained pediatric allergologists. Component resolved diagnosis is subject to considerable complexity and, like many new commercially available diagnostic methods and pharmacological breakthroughs, to both ethical issues and potentially demanding economic consumption of resources which make it necessary to discuss these aspects thoroughly [41]. 

## 9. Conclusions

In several IgE-mediated conditions in children, the use of component resolved diagnosis in the hands of trained clinicians will offer improved diagnosis and therapy. There is evidence for investigating selected cases of suspected peanut allergy, birch pollen allergy, and associated cross-reactivity by means of component analyses. More than 95% of patients with IgE antibodies to Ara h 2 in combination with Ara h 1 or Ara h 3 have symptoms when ingesting peanuts. Whether there may be a threshold level of IgE to Ara h 2, however, above which peanut allergy may be diagnosed with a sufficient clinically sensitivity and specificity which may abandon the need for oral provocation remains to be evaluated. If it is important to know the degree of severity of the allergic reaction to peanut, oral provocation should still be considered. 

 Component resolved diagnosis has a place in the investigation of children with insect allergy. For the frequent cases of an unclear history and increased IgE to both bee and wasp assessment of IgE to Api m 1 and Ves v 5 is helpful for the decision of whether immunotherapy to both allergens should be recommended or not. 

 Component resolved diagnosis may also be indicated in the investigation of rare, anaphylactic reactions in patients with suspected wheat or soy allergy.

 Prospectively planned protocols for clarification of the extent to which component resolved diagnosis will be able to improve selection of patients to immunotherapy and, thus, the efficacy of immunotherapy, are needed. 
